# Color Image Enhancement Focused on Limited Hues

**DOI:** 10.3390/jimaging8120315

**Published:** 2022-11-23

**Authors:** Tadahiro Azetsu, Noriaki Suetake, Keisuke Kohashi, Chisa Handa

**Affiliations:** 1 Department of Culture and Creative Arts, Yamaguchi Prefectural University, Yamaguchi 753-8502, Japan; 2 Graduate School of Sciences and Technology for Innovation, Yamaguchi University, Yamaguchi 753-8512, Japan

**Keywords:** image enhancement, limited hues, hue preservation, CIELAB, sRGB

## Abstract

Some color images primarily comprise specific hues, for example, food images predominantly contain a warm hue. Therefore, these hues are essential for creating delicious impressions of food images. This paper proposes a color image enhancement method that can select hues to be enhanced arbitrarily. The current chroma is considered such that near achromatic colors are not over-enhanced. The effectiveness of the proposed method was confirmed through experiments using several food images.

## 1. Introduction

Color images permeate our daily lives by recording various scenes. The popularity of smartphones and other digital devices has created an environment where people can easily capture photographs. Furthermore, they can share photographs using Internet technologies, such as social networking services (SNSs).

For instance, we can obtain many food images taken in various environments, which are posted on SNSs. However, their quality may be poor because of the performance of the digital device and lighting environment. Food images appear delicious when their lightness and chroma are satisfactory. In addition, food images mainly involve warm hues, such as red, yellow, and orange. Therefore, color image enhancement that focuses on warm hues is necessary for food images. Furthermore, the hues should not have changed after the image enhancement.

Several image enhancement methods that preserve the hue in the RGB color space have been proposed to improve the quality of acquired images [1,2,3,4,5,6,7,8]. Furthermore, image enhancement methods in other color spaces, such as HSI and CIELAB color spaces, have been proposed [9,10,11,12,13,14]. These color spaces can directly represent hue, chroma, and lightness.

Our previous method [14] using the CIELAB color space was computationally expensive and real-time processing was difficult. The improved method [15,16] solved the problem of computational cost; however, its concrete applications in daily life were unclear. We focus on the limited hues that often appear in food images and propose an image enhancement method that preserves the hue in the CIELAB color space to match human visual characteristics. The novelties of the proposed method are that a weighting function with hue as a variable is introduced to achieve limited-hue image enhancement naturally and the unnatural coloring of near-achromatic colors is avoided by considering the magnitude of the current chroma. However, one limitation of the proposed method is that it does not incorporate the local features of the human visual system such as the Abney effect [17] and the Helmholtz–Kohlrausch effect [18,19]. Experiments were conducted using several digital images to verify the performance of the proposed method.

The rest of this paper is organized as follows. Section 2 explains a limited hue-focused chroma enhancement method. Section 3 provides the experimental results applying the proposed image enhancement method to some digital food images. Section 4 presents the conclusions of our study.

## 2. Limited Hue-Focused Chroma Enhancement in CIELAB Color Space

This study employs the CIELAB color space to represent human visual perception adequately as the color space for image enhancement processing.

### 2.1. Conversion from RGB Color Space to CIELAB Color Space

Generally, digital color images acquired using digital devices are represented in the RGB color space. Therefore, the conversion from RGB color components to CIELAB color components is required. The RGB components of the original image are first inverse gamma corrected and converted to the linear RGB components. Here, the RGB color space is assumed to be the sRGB color space. The linear RGB components are denoted as ${\left\{Q_{C}\right\}}_{C\in \{R,G,B\}}$. *R*, *G*, and *B* denote red, green, and blue, respectively. We assume that $\left\{Q_{C}\right\}$ is normalized to [0,1].

Next, $Q_{C}$ is converted into the color components (*X*, *Y*, *Z*) of the CIEXYZ color space. Finally, (X,Y,Z) is converted to color components ($L^{*}$, $a^{*}$, $b^{*}$) of the CIELAB color space. $L^{*}$ is the lightness ranging from 0 to 100. $a^{*}$ and $b^{*}$ are chromaticity indices in the red-green and yellow-blue directions, respectively.

Chroma $C^{*}$ and hue *h* are given by the following equations using $a^{*}$ and $b^{*}$ [20].

(1) $$\begin{array}{cc} C^{*} & =\sqrt{{\left(a^{*}\right)}^{2}+{\left(b^{*}\right)}^{2}}, \end{array}$$

(2) $$\begin{array}{cc} h & =\arctan(b^{*}/a^{*}). \end{array}$$

Although *h* is generally given in the range [0,360], in this study, it is treated as [−180,180] in order to simplify the formulation. The minimum value of $C^{*}$ is zero and the maximum value varies depending on $L^{*}$ and *h*. Here, *h* is set to 0 when $C^{*}<0.1$.

### 2.2. Hue-Based Weight Function for Chroma Enhancement

**Figure 1:**
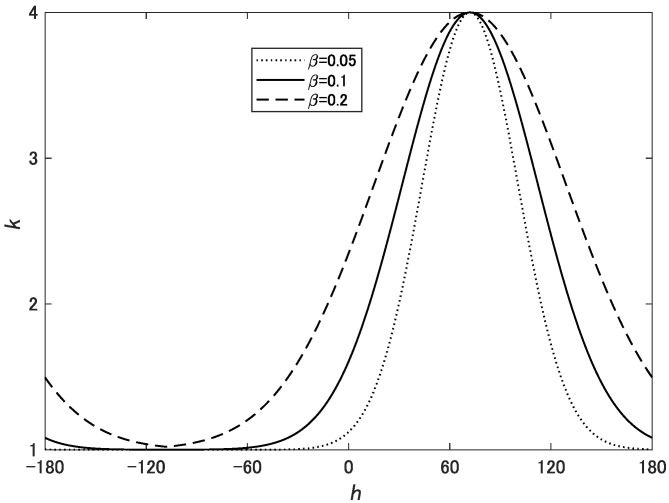
Weight function *k* expressed by Equation (3) for α=3, θ=72, and β=0.05,0.1,0.2.

The weight function *k* is introduced to achieve chroma enhancement in a limited range of hues.

(3) $$\begin{array}{c} k=\alpha \exp\left(-\frac{{\left(\left(h-\theta \right)/180\right)}^{2}}{\beta }\right)+1, \end{array}$$

where α, β, and θ are parameters that determine the shape of Gaussian function. θ is the target hue and is the center of the Gaussian function. α determines the degree of chroma enhancement. β determines the range of hues that need to be enhanced. When β is small, only hues close to θ are enhanced, and when β is larger, wider hues are enhanced. Figure 1 shows the weight function *k*.

Using the weight function *k*, the chroma enhancement while preserving the hue is performed as follows:

(4) $$\begin{array}{c} C_{k}^{*}=\sqrt{{\left(ka^{*}\right)}^{2}+{\left(kb^{*}\right)}^{2}}=kC^{*}. \end{array}$$

When *k* is close to 1, the enhanced chroma $C_{k}^{*}$ is almost the same as that of the original $C^{*}$. *h* is preserved in the CIELAB color space from $\arctan(kb^{*}/ka^{*})$=$\arctan(b^{*}/a^{*})$. However, the RGB color components obtained by converting the enhanced CIELAB color components ($ka^{*}$, $kb^{*}$, $L^{*}$) may be out of the color gamut, depending on the degree of *k*. In this case, the quality of the resulting image degrades.

**Figure 2:**
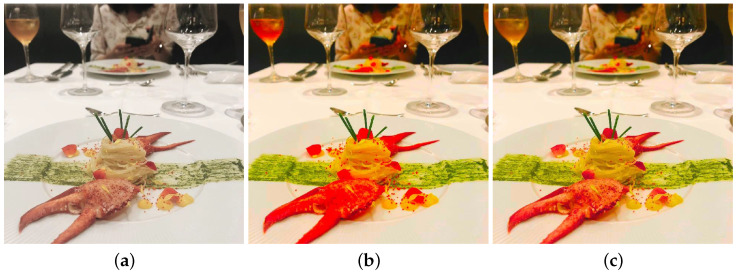
Experimental results. (**a**) Original image. (**b**) Resulting image applying only Equation (3). (**c**) Resulting image applying Equation (5) to (**b**).

To address the color gamut problem, the chroma $C_{e}^{*}$ after enhancement is given as follows:

(5) $$\begin{array}{c} C_{e}^{*}\;=\;\left\{\begin{array}{cc} C_{k}^{*} & \;\mathrm{within}\;\mathrm{gamut}\\ C_{\mathrm{LU}}^{*} & \;\mathrm{otherwise} \end{array}\right.. \end{array}$$

where $C_{\mathrm{LU}}^{*}$ is an approximation of the maximum chroma in the color gamut, defined by $L^{*}$, and *h* and is obtained from a lookup table using the method in Ref. [15]. Finally, the enhanced CIELAB color components are converted into RGB color components to obtain the resulting images [15]. Figure 2 shows the effects of the gamut correction using Equation (5).

### 2.3. Adjustment of the Degree of Chroma Enhancement Considering Current Chroma

**Figure 3:**
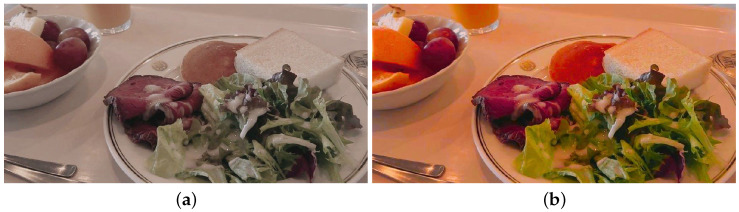
Experimental results. (**a**) Original image. (**b**) Resulting image using the proposed method with Equation (3).

However, as illustrated in Figure 3, there is a problem that unnatural colors are added to colors that are close to achromatic colors, such as whitish colors.

To solve this problem, function *t* is added to Equation (3) such that the degree of chroma enhancement is adjusted according to the magnitude of the current chroma.

(6) $$\begin{array}{c} k=\alpha t\left(C^{*}/C_{\max}^{*}\right)\exp\left(-\frac{{\left(\left(h-\theta \right)/180\right)}^{2}}{\beta }\right)+1, \end{array}$$

where $C_{\max}^{*}$ is the maximum chroma of the original image. *t* is a tone-mapping function defined as follows:

(7) $$\begin{array}{c} t\left(x\right)\;=\;\left\{\begin{array}{cc} 0 & \;x<\mathrm{MIN}\\ \frac{x-\mathrm{MIN}}{\mathrm{MAX}-\mathrm{MIN}} & \;\mathrm{MIN}\leq x\leq \mathrm{MAX}\\ 1 & \;x>\mathrm{MAX} \end{array}\right.. \end{array}$$

**Figure 4:**
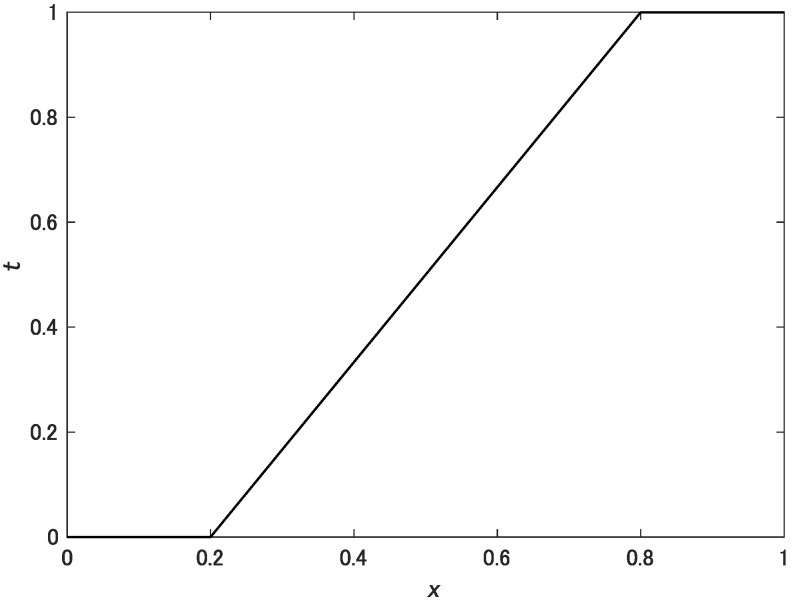
Tone mapping function *t* expressed by Equation (7) for MIN=0.2 and MAX=0.8.

**Figure 5:**
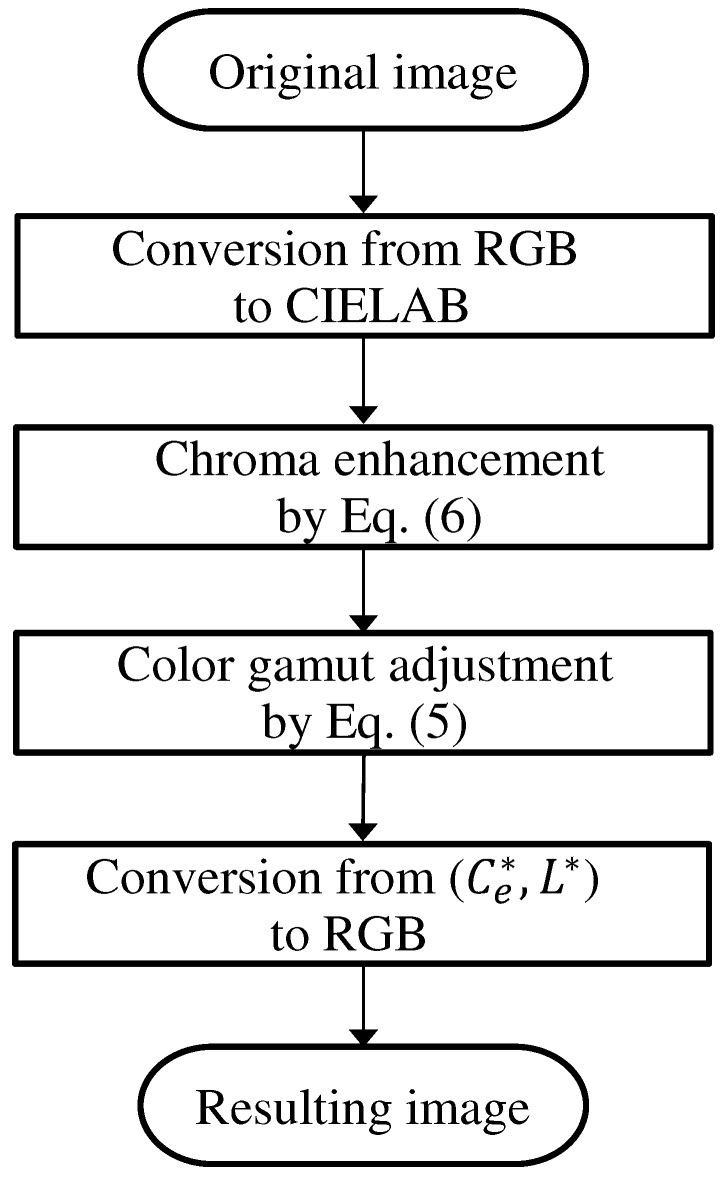
Flow diagram of the proposed method.

Figure 4 shows the tone-mapping function *t*. The proposed method is shown in Figure 5.

## 3. Experimental Results

### 3.1. Food Image Enhancement

Experiments were conducted to illustrate the performance of the proposed method using digital food images. Parameters α, β, MIN, and MAX are set as to 3, 0.1, 0.2, and 0.8, respectively. θ is 72, which is the average value of *h*, using the top 100 ranking photos posted as of April 2021 on the SNS site “SnapDish”, which specializes in cooking [21]. The problem in this calculation is that the values of *h* differ greatly between −180 and 180, even though they have almost the same hue. Therefore, θ is determined using the following equation:

(8) $$\begin{array}{c} \theta =\langle \arctan\left(\bar{b^{*}}/\bar{a^{*}}\right)\rangle , \end{array}$$

where <·> is the average operator for all the images. $\bar{a^{*}}$ and $\bar{b^{*}}$ are the average values of $a^{*}$ and $b^{*}$ in each image.

**Figure 6:**
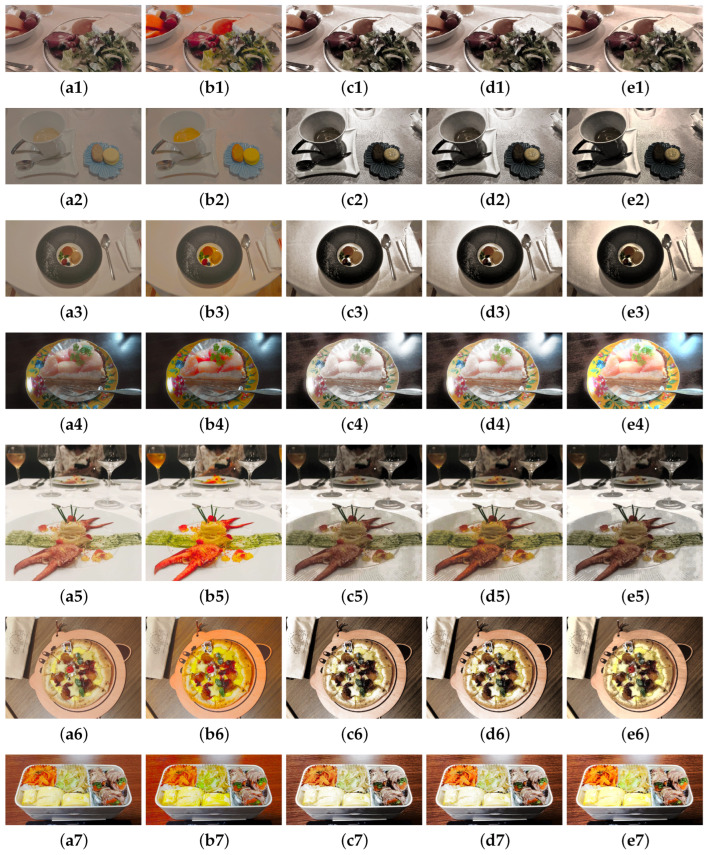
Experimental results. First column (**a1–a7**): original images. Second column (**b1–b7**): resulting images using the proposed method with Equation (6). Third column (**c1–c7**): resulting images using the method in Ref. [2] using histogram equalization. Fourth column (**d1–d7**): resulting images using the method in Ref. [6] using histogram equalization. Fifth column (**e1–e7**): resulting images using the method in Refs. [12,13].

Figure 6 shows the experimental results of seven digital images. These are 24-bit color images (1) 1015 × 501 pixels, (2) 1920 × 1080 pixels, (3) 1919 × 1080 pixels, (4) 1706 × 960 pixels, (5) 1280 × 1280 pixels, (6) 1478 × 1108 pixels, and (7) 1280 × 664 pixels in size, respectively. The first column shows the original images, the second column shows the resulting images using the proposed method, and the third, fourth, and fifth columns show the resulting images using the methods in Ref. [2], in Ref. [6], and in Refs. [12,13]. In (b1), the unnatural coloring of whitish colors is improved compared with (b) in Figure 3. In (b2) and (b3), the chroma of the sweets is appropriately enhanced, whereas the blue and gray dishes remain nearly identical. In (b4) and (b5), the cake and crab are vivid, whereas the brown and white tables are largely unaffected. In (b6) and (b7), the yellow areas are effectively brightened. In addition, we can see that the proposed method provides sufficient color enhancement compared to the comparison methods. However, the resulting image of (e4) is vivid compared with (b4), which was obtained by the proposed method. In the experiments, the same parameters were used for all images. This was done to demonstrate the versatility of the proposed method. However, to further improve the performance of the proposed method, it is necessary to consider a scheme by changing the parameters based on the statistics of the input image.

The difference in hue between the resulting and original images is calculated as follows [22].

(9) $$\begin{array}{c} ||\Delta h^{*}\left|\right|=2\sqrt{C_{e}^{*}C_{o}^{*}}\sin\left(\frac{|h_{e}-h_{o}|}{2}\right), \end{array}$$

where $h_{e}$ and $h_{o}$ are the hues of pixels paired with the resulting and the original images, respectively. Similarly, $C_{e}^{*}$ and $C_{o}^{*}$ represent chromas. $\left|\cdot \right|$ denotes the absolute value. Furthermore, the difference $\Delta C^{*}=C_{e}^{*}-C_{o}^{*}$ is calculated to verify the degree of image enhancement. Table 1 lists the averages and standard deviations of $||\Delta h^{*}\left|\right|$ and $\Delta C^{*}$. We can see that the averages of $||\Delta h^{*}\left|\right|$ in all resulting images by the proposed method are smaller than those of the comparison methods, and the hue is almost preserved. Furthermore, the averages of $\Delta C^{*}$ indicate that the proposed method can improve the chroma compared to the comparison methods.

SSIM [23] is measured as the image quality metric for objectively evaluating the performance of the proposed method. When SSIM is closer to 1, the enhanced image is structurally similar to the original image. Table 2 shows SSIMs between the original images and the resulting images with respect to Figure 6. This value is the average of SSIM calculated for each RGB component. From Table 2, we see that the proposed method gives better results compared to the comparison methods.

**Figure 7:**
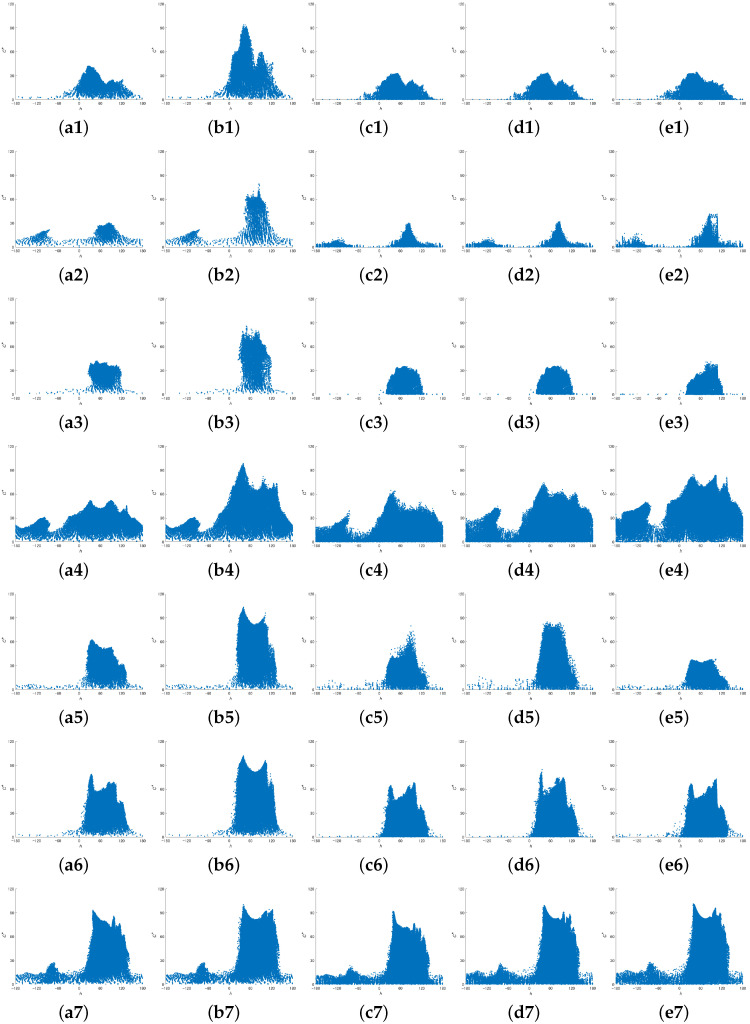
Scatter plots of hue and chroma corresponding to Figure 6.

Figure 7 shows the scatter plots of the hue and chroma corresponding to Figure 6. The chroma is enhanced within a limited range of hues by the proposed method. However, due to the relationship between the RGB color space and the CIELAB color space, the range of possible values for $C^{*}$ varies depending on $L^{*}$. Therefore, there are areas where $C^{*}$ does not increase uniformly.

Furthermore, Scheffe’s paired comparison test [24,25] was conducted as a subjective evaluation of the resulting images in Figure 6. Randomly selected images were placed left and right, and the two images were evaluated by six examinees (average age: 20.5 years, 4 females and 2 males) about which image is preferable as a food image. The examinees evaluated the two images by selecting one of [−2,−1,0,1,2], where the higher values indicate that the right image is preferable to the left image. The yardstick method was used to obtain the evaluation value of each image. Table 3 shows the evaluation values by Scheffe’s paired comparison test. A higher value indicates a higher evaluation of the image. From Table 3, we see that the proposed method is superior to the comparison methods except in the case of image 4.

### 3.2. Other Applications

**Figure 8:**
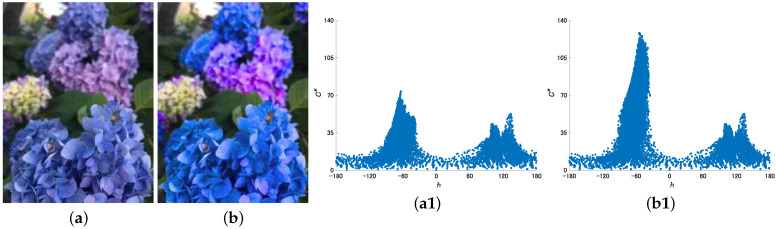
Experimental results. (**a**) Original image and its scatter plot of hue and chroma (**a1**). (**b**) Resulting image using the proposed method with Equation (6) and its scatter plot of hue and chroma (**b1**).

Figure 8 shows the experimental results of the flower image. As for the values of the parameters, only θ was changed to −60 due to the color information of the original image. This figure shows that the proposed method can naturally highlight almost only the color of the blue flowers.

### 3.3. Computational Load

Table 4 shows the execution times required to obtain Figure 6 using CPU Intel(R) Core(TM) i5-5200U, RAM 8GB, MATLAB R2020a. The computational cost of the proposed method is relatively high because of the color space conversion involved. However, since the implementation of the proposed method is still naive, we think that further acceleration is fully possible. The Matlab code of the proposed method is available here: https://github.com/ta850-z/limited_hues_enhancement, accessed on 20 November 2022.

## 4. Conclusions

This paper proposed a color image enhancement method that focuses on limited hues. The degree of chroma enhancement was also adjusted by considering the magnitude of current chroma to avoid the unnatural coloring of near-achromatic colors. Applying the proposed method to actual food images confirmed that the image enhancement is effective. Further work is required to improve the proposed method by utilizing the color information of the original image. 

## Figures and Tables

**Table 1:** Averages and standard deviations of $||\Delta h^{*}\left|\right|$ and $\Delta C^{*}$.

		$||\Delta h^{*}\left|\right|$		$\Delta C^{*}$	
		Ave	Std	Ave	Std
Proposed method using Equation (6)	(b1)	0.003	0.033	8.186	10.942
	(b2)	0.001	0.003	3.849	7.585
	(b3)	0.001	0.023	5.063	6.331
	(b4)	0.005	0.051	4.202	7.976
	(b5)	0.002	0.021	7.449	14.096
	(b6)	0.004	0.070	13.136	11.835
	(b7)	0.020	0.160	10.727	12.332
The method in Ref. [2] using histogram equalization	(c1)	0.104	0.218	−4.558	3.517
	(c2)	0.057	0.074	−4.124	3.367
	(c3)	0.078	0.120	−4.154	3.315
	(c4)	0.364	0.728	−3.982	8.227
	(c5)	0.084	0.135	−2.096	6.152
	(c6)	0.227	0.393	−7.997	6.841
	(c7)	0.517	0.996	−6.995	6.180
The method in Ref. [6] using histogram equalization	(d1)	0.104	0.218	−4.546	3.533
	(d2)	0.054	0.068	−4.007	3.446
	(d3)	0.087	0.118	−3.937	3.551
	(d4)	0.266	0.586	−0.143	8.605
	(d5)	0.263	0.584	0.764	7.378
	(d6)	0.305	0.417	−6.947	8.024
	(d7)	0.369	0.700	−4.943	7.690
The method in Refs. [12,13]	(e1)	0.434	1.329	−0.961	3.029
	(e2)	0.411	1.259	−1.130	4.859
	(e3)	0.473	1.335	−1.492	4.246
	(e4)	1.112	2.783	7.764	8.496
	(e5)	0.093	0.260	−4.449	5.866
	(e6)	0.770	1.776	−3.361	5.128
	(e7)	0.476	1.327	0.666	6.274

**Table 2:** SSIMs between the original images and the resulting images with respect to Figure 6.

		b	c	d	e
Number	1	0.939	0.714	0.714	0.751
	2	0.973	0.341	0.341	0.300
	3	0.973	0.576	0.576	0.480
	4	0.952	0.468	0.469	0.378
	5	0.925	0.607	0.603	0.580
	6	0.896	0.760	0.761	0.806
	7	0.881	0.841	0.835	0.848

**Table 3:** Evaluation values by Scheffe’s paired comparison test with respect to Figure 6.

		b	c	d	e
Number	1	1.167	−0.625	−0.708	0.167
	2	0.750	−0.375	−0.417	0.042
	3	1.042	−0.250	−0.125	−0.667
	4	0.250	−0.875	−0.250	0.875
	5	0.708	−0.375	0.417	−0.750
	6	0.625	−0.375	−0.292	0.042
	7	0.917	−0.667	−0.750	0.500

**Table 4:** Execution times (s) required to obtain Figure 6.

		b	c	d	e
Number	1	1.69	0.25	0.33	2.71
	2	6.25	0.69	1.10	21.04
	3	6.26	0.68	1.01	21.07
	4	5.10	0.60	0.84	14.89
	5	5.11	0.64	0.90	19.47
	6	5.00	0.62	0.87	16.99
	7	2.64	0.36	0.49	5.59

## Data Availability

Not applicable.
